# Supplementary material for: The Prevalence of Small for Gestational Age and Extrauterine Growth Restriction among Extremely and Very Preterm Neonates, Using Different Growth Curves, and Its Association with Clinical and Nutritional Factors
Source: Nutrients. 2023 Jul 25;15(15):3290. doi: 10.3390/nu15153290 (PMC10420820; doi:10.3390/nu15153290)
Supplement: Supplementary file 1 [file nutrients-15-03290-s001.zip › nutrients-2509228-supplementary.pdf]

Supplementary Table S1. Associations* of perinatal, clinical and nutritional factors with weight at discharge z scores based on the Fenton2013 and INTERGROWTH-21st weight growth references, stratified by year of hospitalization (before or after 2018)

	Before 2018 (n=274)		After 2018 (n=163)	
	**Fenton2013 β (95% CI)**	**INTERGROWTH-21st β (95% CI)**	**Fenton2013 β (95% CI)**	**INTERGROWTH-21st β (95% CI)**
**Perinatal factors**				
Extremely preterm	0.58 (0.19,0.96)	0.43 (0.03,0.83)	0.64 (0.08,1.20)	0.73 (0.12,1.33)
BW z score	0.29 (0.16,0.41)	0.40 (0.27,0.53)	0.51 (0.34,0.68)	0.49 (0.33,0.65)
Hospitalization (days)	-0.04 (-0.05,-0.03)	-0.04 (-0.05,-0.03)	-0.04 (-0.05,-0.03)	-0.03 (-0.04,-0.02)
**Nutritional factors**				
PN duration (days)	0.01 (-0.01,0.02)	0.01 (-0.01,0.03)	0.01 (-0.00,0.03)	0.00 (-0.01,0.02)
EN initiation (days)	-0.03 (-0.07,0.01)	-0.04 (-0.08,0.00)	-0.07 (-0.15,0.02)	-0.08 (-0.17,0.01)
Full EN achieved (days)	-0.01 (-0.03,0.00)	-0.02 (-0.04,0.00)	-0.02 (-0.05,0.00)	-0.02 (-0.05,0.00)
**Clinical factors**				
BPD	0.47 (-0.08,1.02)	0.61 (0.04,1.17)	0.12 (-0.38,0.62)	0.24 (-0.29,0.78)
Late-onset Sepsis	-0.03 (-0.32,0.27)	-0.08 (-0.38,0.22)	-0.15 (-0.52,0.22)	-0.22 (-0.62,0.17)
Anemia	0.42 (0.11,0.73)	0.36 (0.04,0.67)	0.26 (-0.10,0.63)	0.27 (-0.13,0.66)
hsPDA	-0.04 (-0.51,0.43)	-0.02 (-0.50,0.47)	-0.85 (-1.36,-0.34)	-0.77 (-1.31,-0.23)
ROP	0.38 (-0.29,1.06)	0.37 (-0.33,1.07)	0.04 (-0.63,0.71)	-0.02 (-0.74,0.69)
Respiratory support (days)	0.00 (-0.02,0.01)	0.00 (-0.02,0.01)	0.02 (-0.00,0.04)	0.01 (-0.01,0.04)
Oxygen administration (days)	0.01 (-0.00,0.02)	0.01 (-0.00,0.02)	-0.01 (-0.03,0.01)	-0.01 (-0.03,0.01)

Abbreviations: SGA, small for gestational age; PN, parenteral nutrition;EN, enteral nutrition; BPD, bronchopulmonary dysplasia; hsPDA, hemodynamically significant patent ductusarteriosus; ROP, retinopathy of prematurity (⩾Stage II); cystic PVL, cystic periventricular leukomalacia. * Beta coefficients (β) corresponding confidence intervals (CI) obtained using linear regression models, respectively.

Supplementary Table S2. Associations* of perinatal, clinical and nutritional factors with extrauterine growth restriction, based on the Fenton2013 and INTERGROWTH-21st weight growth references, stratified by year of hospitalization (before or after 2018)

	**Before 2018 (n=274)**		**After 2018 (n=163)**	
	**Fenton2013 OR (95% CI)**	**INTERGROWTH-21st OR (95% CI)**	**Fenton2013 OR (95% CI)**	**INTERGROWTH-21st OR (95% CI)**
**Perinatal factors**				
Extremely preterm	0.84 (0.25,2.84)	0.69 (0.20,2.32)	0.01 (0.00,0.23)	0.14 (0.01,1.70)
BW z score	0.20 (0.11,0.34)	0.19 (0.11,0.34)	0.12 (0.05,0.27)	0.29 (0.16,0.51)
Hospitalization (days)	1.07 (1.04,1.10)	1.08 (1.05,1.12)	1.08 (1.03,1.13)	1.06 (1.02,1.10)
**Nutritional factors**				
PN duration (days)	0.97 (0.91,1.02)	0.95 (0.89,1.01)	1.17 (1.00,1.37)	1.11 (0.99,1.26)
EN initiation (days)	1.09 (0.97,1.22)	1.23 (1.08,1.40)	1.24 (0.97,1.58)	1.25 (0.96,1.64)
Full EN achieved (days)	1.08 (1.03,1.14)	1.04 (0.99,1.10)	0.90 (0.77,1.05)	0.94 (0.84,1.05)
**Clinical factors**				
BPD	0.56 (0.12,2.64)	0.57 (0.11,3.01)	1.30 (0.22,7.77)	2.10 (0.33,13.44)
Late-onset Sepsis	1.99 (0.82,4.82)	2.49 (1.00,6.20)	0.34 (0.08,1.47)	3.07 (0.77,12.31)
Anemia	0.30 (0.12,0.77)	0.32 (0.11,0.92)	0.62 (0.18,2.16)	0.90 (0.20,4.10)
hsPDA	1.45 (0.29,7.39)	0.48 (0.10,2.31)	7.36 (1.21,44.84)	2.83 (0.49,16.43)
ROP	0.20 (0.02,1.73)	0.10 (0.01,1.13)	na	7.10 (0.53,94.91)
Respiratory support (days)	0.99 (0.94,1.04)	1.02 (0.97,1.06)	0.97 (0.90,1.05)	0.99 (0.92,1.07)
Oxygen administration (days)	0.97 (0.93,1.01)	0.98 (0.94,1.02)	1.04 (0.98,1.12)	0.99 (0.93,1.05)

Abbreviations: SGA, small for gestational age; PN, parenteral nutrition;EN, enteral nutrition; BPD, bronchopulmonary dysplasia; hsPDA, hemodynamically significant patent ductusarteriosus; ROP, retinopathy of prematurity (⩾Stage II); cystic PVL, cystic periventricular leukomalacia. * Odds ratios (OR) and corresponding confidence intervals (CI) obtained using logistic regression models, respectively.
